# Antifungal chemotherapies and immunotherapies for the future

**DOI:** 10.1111/pim.12960

**Published:** 2022-12-18

**Authors:** Darius Armstrong‐James

**Affiliations:** ^1^ Department of Infectious Diseases Medical Research Council Centre for Molecular Bacteriology and Infection, Imperial College London London UK

**Keywords:** fungi, immunotherapies, novel, therapies

## Abstract

Human fungal pathogens cause a broad plethora of infections, spanning cutaneous dermatophytoses to invasive infections in immunocompromised hosts. As eukaryotic pathogens are capable of morphotype switching, they present unique challenges both for drug development and the immunological response. Whilst current antifungal therapies are limited to the orally available triazoles, intravenous echonocandins and polyenes, and flucytosine and terbinafine, there has been recent significant progress in the antifungal armamentorium with ibrexafungerp, a novel orally available terpanoid that inhibits 1,3‐beta‐D‐glucan‐approved by Food and Drug Administration in 2021, and fosmanogepix, an orally available pro‐drug of manogepix, which targets glycosylphosphatidylinositol‐anchored protein maturation entering Phase 3 studies for candidaemia. A number of further candidates are in development. There has been significant use of existing immunotherapies such as recombinant interferon‐γ and G‐CSF for fungal disease in immunocompromised patients, and there are emerging opportunities for monoclonal antibodies targeting TH2 inflammation. Omalizumab, an anti‐IgE monoclonal antibody in asthma, is now used routinely for the treatment of allergic bronchopulmonary aspergillosis, and further agents targeting IL‐4 and IL‐5 are being evaluated. In addition, T‐cell CAR therapy is showing early promise for fungal disease. Thus, we are likely to see rapid advances to our approach to the management of fungal disease in the near future.

## 1 INTRODUCTION

Human fungal pathogens cause a broad plethora of infections in both the immunocompetent and immunocompromised host. Primary fungal infections range from dermatophytoses such as athlete's foot and tinea corporis, causing cutaneous infections at some point in a third of the global population, to invasive endemic mycoses such as histoplasmosis, talaromycosis and sprototrichosis that are usually geographically restricted and primarily cause pulmonary or deep cutaneous infection.^1^ Opportunistic fungal infections such as aspergillosis, candidiasis and cryptococcosis are a major unsolved problem across healthcare settings, causing invasive or chronic, difficult to diagnose and difficult to treat, life‐threatening infections across a broad range of immunocompromised, critical care and respiratory disease patients.^2^, ^3^, ^4^ There are fundamental aspects of fungal pathogens that make them a particular clinical problem; for instance, the ability to transition from single‐cell infecting forms to multicellular mycelial networks within the host presents challenges for both the immune system and antifungal chemotherapies.^5^ The fact that fungi, like us, are eukaryotic organisms, presents a further challenge, as it restricts the number of targets for drug development that do not have human homologues.^6^ Furthermore, as fungi are elite saprophytes in nature, they deploy a bewildering array of proteases and secondary metabolites that can further enable tissue destruction at the site of infection and disable the consequential immunological response.^7^, ^8^ Taken together, these considerations present a huge challenge for modern medicine, which is borne out in the generally poor responsiveness of fungal infections and diseases to currently available treatments.^9^ Whilst great progress has been made in understanding the ways in which fungi can interact with other microbes and plants in mycorrhizial networks and lichens,^10^ for instance, we are only at the beginning of our journey to understand the implications of fungal symbiosis with other microbes in the human microflora.^11^ As the future therapeutic landscape for human fungal diseases unfolds, understanding these complex interactions will clearly be of great importance.

## 2 CURRENT THERAPEUTIC APPROACHES TO FUNGAL DISEASE

The current arsenal of antifungal therapies is restricted, with only three major drug classes available as first‐line therapies. The polyenes (targeting fungal ergosterol), such as amphotericin B and nystatin, have been available since the 1950s, and have a remarkable wide spectrum of activity against most fungal pathogens; furthermore, antifungal resistance to this class of drugs has not significantly emerged during this time, although there are some fungal species with intrinsic resistance, such as *Aspergillus terreus* and *Candida lusitaneae*.^12^ There are however a number of limitations to this drug class, in that they are only currently available as intravenous formulations or as topical agents. Furthermore, there is significant toxicity associated with the polyenes, typically amphotericin B deoxycholate, when given intravenously, with nephrotoxicity being a particular issue due to drug microaggregate formation in the loop of Henle.^12^ This has led to the development of a number of lipid‐based formulations that hold the drug in its monomeric form, of which liposomal amphotericin B is the best‐known example.

The triazoles and imidazole derivatives that also target ergosterol synthesis have the advantage of a better toxicity profile and, importantly, are available orally. Fluconazole has specific utility for *Candida albicans* and *Cryptococcus neoformans* but no activity for *Aspergillus* species and patchy utility across dermatophytes and endemic mycoses.^13^ Itraconazole and, more recently, posaconazole are lipophilic triazoles that enable a broader spectrum of activity against most mould species, while voriconazole is an aquaphilic derivative of fluconazole engineered specifically for activity against *Aspergillus* species with less activity against other moulds.^14^ Isavuconazole is the most recent triazole to gain licensing; it is an aquaphilic triazole with structural similarity to voriconazole but with broad activity and reduced interactions.^15^

The echnocandins (caspofungin, anidulafungin and micafungin), semi‐synthetic pneumocandin derivatives, are intravenously formulated and have broad‐spectrum activity against *Candida* species, with some activity against *Aspergillus* species.^16^ They have an excellent safety and tolerability profile, and significant resistance has not emerged except in *Candida glabrata* and *auris*. They target beta‐1,3‐D glucan synthase, inhibiting the production of beta‐1,3‐D glucan, an essential fungal cell wall component.

Other currently available antifungals include terbinafine, an ergosterol inhibitor with good oral bioavailability and activity against dermatophytes and dematiaceous (black) moulds,^17^ and flucytosine, an orally and intravenously available flurouracil pro‐drug that is semi‐selective for inhibition of fungal purine synthesis and mostly limited to adjunctive use due to the rapid emergence of resistance.^18^

## 3 CURRENT DRUGS IN THE ANTIFUNGAL PIPELINE

Fosmanogepix (APX001, Amplyx Pharamceuticals Inc., San Diego, CA) is an orally and intravenously available broad‐spectrum antifungal.^19^ Systemic phosphatases rapidly convert it to manogepix. The company has recently been acquired by Pfizer. Manogepix targets glycosylphosphatidylinositol‐anchored protein maturation by inhibition of the fungal enzyme Gwt1, an inositol acetyl transferase that is critical for the trafficking of mannoproteins to the fungal cell wall.^20^ Manogepix has a good spectrum of activity against *Candida* species, *Cryptococci* spp., *Coccidiodes*, *Aspergillus* spp. and other rare moulds.^19^ In Phase 1 studies, fosmanogepix was well tolerated with no significant events. Phase 2 studies have been undertaken in patients with candidaemia, with a success rate of 80% in the MITT population. A Phase 2 study evaluating fosmanogepix in the treatment of *Candida auris* has also been completed, and ongoing studies are evaluating fosmanogepix for the treatment of invasive aspergillosis and other rare mould infections (NCT04240886). A Phase 3 study is planned to start in December 2022 evaluating safety and efficacy in candidaemia (NCT05421858), and fosmanogepix has been given fast‐track status by the FDA.

Ibrexafungerp is a novel terpanoid that inhibits 1,3‐beta‐D‐glucan, a major constituent of the fungal cell wall.^21^ As such, it is the first orally available glucan synthase inhibitor, and distinct from the echinocandins. It is also fungicidal to *Candida* spp. whilst being fungistatic to *Aspergillus* spp.^22^ Cross‐resistance between these classes does not appear to usually be an issue in *Candida* spp. Oral availability is good, and intravenous formulations are being developed. Animal models show good tissue penetration although central nervous system penetration is poor.^23^ In vitro activity is mainly for *Candida* and *Aspergillus* spp. consistent with echinocandins, with minimal acivity against *Mucorales* spp.^24^ Phase 1/2 studies indicate good tolerability and safety profiles. Phase 2 studies indicated similar efficacy to echinocandins or fluconazole for the treatment of candidiasis, and there are now several Phase 3 studies in play.^25^, ^26^ There are two open labels, single arm studies addressing candidiasis and aspergillosis. Ibrexafungerp was approved by the FDA on 1st June 2021 for the treatment of vulvovaginal candidiasis based on two Phase 3 studies, VANISH 303 and 306.^27^ There is an ongoing study of combination therapy with voriconazole for aspergillosis (SYCNERGIA. NCT03672292) and a further study addressing refractory fungal infection (NCT03059992).

Olorofim is an inhibitor of the fungal enzyme dihydroorotate dehydrogenase and interferes with pyrimidine synthesis.^28^, ^29^ It has good tissue distribution but is highly protein‐bound with poor aqueous solubility. Oral biovailability is 45%, and the drug is susceptible to CYP450 enzymes but does not affect them itself.^30^ Olorofim does not have a broad spectrum of antifungal activity but rather specific activity against *Aspergillus* spp. and *Fusarium* spp., endemic mycosis and demetiaceous moulds such as *Scedosporium* spp. and *Lometospora* spp.^31^ In terms of clinical studies, olorofim appeared safe in Phase 1 studies, although there were significant infusion‐related reactions.^9^ Olorofim is currently being evaluated clinically through an open‐label Phase 2b trial focused on patients with limited treatment options for invasive fungal infections (NCT03583164). A further Phase 3 randomized study comparing the efficacy of olorofim to ambisome in invasive aspergillosis is planned (NCT05101187). In June 2022, the FDA granted olorofim qualified infectious disease product designation for the treatment of selected invasive fungal diseases.

Opelconazole is a broad‐spectrum triazole antifungal developed specifically for inhalational therapy.^32^ It is designed for accumulation in the lungs without significant systemic exposure, thus minimizing toxicities and interactions with other therapies through the CYP450 system.^33^ Consistent with other triazoles, it inhibits lanosterol 14α‐demethylase (CYP51A1) thereby blocking ergosterol synthesis and cell membrane formation. It has a broad spectrum of activity against *Aspergillus* spp. apart from *Aspergillus niger*, *Candida* spp. and *Rhizopus oryzae*.^34^, ^35^, ^36^ It has synergistic activity with systemic triazoles in murine models of pulmonary aspergillosis. Phase 1/2 studies in healthy individuals and asthmatics indicate good tolerability with minimal systemic exposure. An ongoing compassionate use programme has shown promising results with successful salvage in eight of the nine patients treated,^37^ and an open‐label Phase 2b study of prophylaxis and pre‐emptive treatment in lung transplant recipients is currently recruiting (NCT05037851). A further double‐blind, placebo‐controlled study has opened addressing refractory pulmonary aspergillosis (NCT05238116). Further drugs targeting Cyp51a include VT1598, which has completed Phase 1 studies, and VT‐1161/Oteseconazole, a novel lanesterol demethylase inhibitor that has shown efficacy for vulvovaginal candidiasis^38^, ^39^ and onychomycosis^40^ in Phase 2 studies.

Rezafungin is a long‐acting intravenous echinocandin. It inhibits beta‐1,3‐D glucan synthase, blocking the production of cell wall beta‐1,3‐D glucan.^41^ A Phase 3 study of candidiaemia and invasive candidiasis has been completed (ReSTORE, NCT03667690), and a further Phase 3 study of invasive fungal infection is ongoing (ReSPECT, NCT04368559).^42^, ^43^ It has broad activity against *Candida* spp. as well as common dermatophytes.^44^, ^45^, ^46^, ^47^ Good efficacy was demonstrated in murine models of invasive candidiasis. It also has some activity against *Aspergillus* spp. and compared favourably to amphotericin B in murine models of disseminated aspergillosis.^48^ However, as is already known for this class, activity against non‐*Aspergillus* moulds and *Cryptococcus neoformans* is limited.

Notably, more recently, MAT2203 or encochleated oral amphotericin B (cAMB) has been developed. This is a lipid nano‐crystal formulation designed for targeted oral delivery of the antifungal drug AMB for the treatment of fungal and parasitic infections.^49^ MAT2203 is currently being assessed for the treatment of vulvovaginal candidiasis (NCT02971007), mucocutaneous candidiasis (NCT02629419) and cryptococcal meningitis (NCT04031833).

## 4 CURRENT AND EMERGING IMMUNOTHERAPEUTIC OPTIONS FOR FUNGAL DISEASE

The majority of invasive fungal infections occur in immunocompromised hosts, such as those with advanced HIV disease, neutropaenia, haematological and solid organ transplant and chronic respiratory diseases.^4^, ^50^, ^51^ A key determinant of outcome from infection is whether or not the host immune response can be reconstituted during antifungal therapy, either by treating the underlying disease, modification of immunosuppressant therapies or through immunotherapeutic approaches.^52^, ^53^ A key principle is to direct the immunotherapy according to the particular immune defect that is thought to be leading to susceptibility to fungal infection.^53^ Therefore, the development of better clinical immunological assays to define fungal immunotherapies in our at‐risk patient groups is a key priority.

Recombinant cytokines have an established role as immunotherapies for fungal infection.^54^, ^55^ Colony stimulating factors such as GM‐CSF and G‐CSF having been used to target both neutrophil activation and myeloid cell population recovery in the context of haematological malignancies in particular.^56^, ^57^ This approach has been shown to have benefit for mucosal and invasive candidiasis, with further case reports supporting its use for invasive aspergillosis and cryptococcal meningoencephalitis.^58^, ^59^, ^60^ G‐CSF is routinely used to increase neutrophil numbers in neutopaenia, a key risk factor for invasive fungal disease.^61^ Randomized controlled studies support its use in invasive candidiasis.^62^ Interferon‐γ, a key antifungal cytokine, enhances macrophage and neutrophil activity, and has been licenced for use as prophylaxis in chronic granulomatous disorder.^63^ It has also been used effectively in the context of invasive candidiasis, organ transplant fungal disease and HIV‐associated cryptococcal meningitis.^60^, ^64^

Immunomodulating therapies are also incrementally important in the context of allergic fungal diseases such as allergic bronchopulmonary aspergillosis, which is characterized by eosinophilic inflammation and TH2/17 responses.^65^ A number of monoclonal antibody therapies targeting IgE (such as omalizumab) or eosinophilic inflammation (such as mepolizumab, an anti‐IL5 monoclonal antibody) have been developed for severe asthma but have utility in ABPA.^66^ The accumulating data for omalizumab are encouraging, with mepolizumab also showing clinical utility, although formal randomized studies in the ABPA have not been undertaken.^67^, ^68^ It should be noted that infection susceptibility is a specific issue for eosinophil‐targeting agents, and thus concomitant antifungals are often also used where there is evidence of fungal airway colonization or infection. There is an ongoing study of the utility of dupilumab, an IL‐4 monoclonal, in ABPA.^69^

The is growing interest in the potential for fungal vaccines and antibodies. For vaccines, a major consideration is that many at‐risk individuals are immunocompromised and thus would not be expected to mount a protective immune response. However, for those undergoing immunosuppressive therapies or being assessed for transplantation there is a window of opportunity in which protective vaccination could be achieved. A further area for advancement is fungal desensitization for allergic fungal diseases, where proof of principle has been established for allergic fungal sinusitis.^70^, ^71^ A major concept for fungal vaccination that has accumulated scientific evidence is to combine antigenic peptides with cell wall pathogen‐associated molecular core patterns, typically mannans and glucans in the context of fungal pathogens.^72^, ^73^ Whilst most data to date are from animal models, there have been Phase 1/2 studies of *Candida* adhesins that established proof of principle.^74^

Cellular therapy with chimeric antigen receptor T cells is rapidly establishing a role for the treatment of haematological malignancies.^75^ This approach allows reprogramming of T cells to have activity against specific antigens, enabling affected cell types to be targeted for killing. Such an approach was adapted using Dectin 1 to engineer cytotoxic T cells capable of killing fungal pathogens in vitro and in animal models of aspergillosis.^76^ A significant draw back for CAR T cell therapy is cytokine storm or risk of alloreactivity with the host.^77^ Innate cellular therapy is also a promising avenue for invasive fungal infection with a long‐established paradigm of granulocyte transfusions in neutropaenic host, typically in the context of aplastic anaemia.^78^ However, this approach has been hindered by the logistical considerations required to deliver effective clinical trials, as granulocytes are short lived. Further avenues being explored include fungal nucleic acid pulsed dendritic cells, although clinical studies have not been undertaken as yet.

## 5 CONCLUSIONS

Management of fungal infection is increasingly complex, given the rapid expansion of immunosuppressive therapies predisposing individuals to life‐threatening infections and the emergence of novel fungal pathogens and antifungal drug resistance. However, the development of a range of novel antifungal therapies, as well as an increasing array of immunomodulatory therapeutics in the clinical arena, has enabled many new options for management. A key challenge for the future will be to personalize approaches and understand the potential for combination therapies in this setting.

## FUNDING INFORMATION

Darius Armstrong‐James is funded by the Wellcome Trust, MRC, Cystic Fibrosis Trust and National Institute of Health Research.

## CONFLICT OF INTEREST

Darius Armstrong‐James declares share options in Pulmocide Ltd., and speaker fees from Gilead Inc.

6

### PEER REVIEW

The peer review history for this article is available at https://publons.com/publon/10.1111/pim.12960.
